# Recent Novelties in Sick-Room Appliances

**Published:** 1886-11-27

**Authors:** 


### Recent Novelties in Sick-Room Appliances.

Hospital Novelties.

The exhibits at the conversazione of the Hospitals Association were a novel feature and of decided interest, some of the sick-room appliances especially attracting much attention. Mr. Pietro Michelli sent from one of the wards of St. Mary's a pretty little cot with tented canopy—his own device, we believe—beneath which reposed the effigy of a little sufferer with bandaged head. Dr. Steele, of Guy's, sent a cot of neat design, also a surgical dressing-table, a self-propelling chair, patent combined coal-bunker and sofa,—a very excellent idea well carried out,—and a special table for the making plaster-of-Paris splints. There were two or three new things in ward lockers, especially noteworthy being a combined locker and seat, exhibited by Messrs. Jenks and Wood. Messrs. Krohne and Sesemann showed a most ingeniously contrived elevating bedstead, which has, we believe, already found its way into Guy's and Middlesex. The elevating bed, which is quite independent of a fixed one beneath it, enables a patient under examination to be placed with the utmost ease and rapidity in any position required. Mr. Newton Nixon of University College, exhibited a tracheotomy or bronchitis kettle, which obviates the necessity for waterproof sheeting over the bed. Messrs. Mayer and Meltzer showed some sanitary and invalid appliances, including a useful bed-rest with foot support, and another with sliding arms. The firm have also devised a table with steel legs, which can be adjusted to the frame of any ordinary bed or cot, the steel legs giving considerable play. Mrs. Bluett, the lady superintendent of Fitzroy House, exhibited the Teignmouth carrying chair, a simple and inexpensive contrivance, consisting of a couple of short poles, and a strap of stout carpet, forming a comfortable seat on which a patient may be carried up and down stairs with the utmost ease. Messrs. Bayley had one of their adjustable tables, which materially facilitates the luxury of breakfasting in bed. Miss Mattocks, of the Cottage Hospital, Oswestry, exhibited some iron splints for compound fracture of leg or thigh, to facilitate the dressing of extensive wounds, which attracted a good deal of attention. Messrs. Robinson and Sons, of Chesterfield, sent some of the late Mr. Sampson Gamgee's patent sponges and dressings. Messrs. Gainsford and Co. displayed some hospital furniture. A very serviceable screen was sent from St. Mary's, which possesses, apparently, the advantage of indestructibility.

Of Interest to Asylum Superintendents.

Messrs. Deverell Bros. showed some mechanical toys, which attracted a crowd of visitors. A fashionably attired little doll—whose hat was of marvellous dimensions—played *La boulangère* to the perfect satisfaction of her auditory. Another toy represented a burning building, up which the firemen climbed on a ladder; while at a neighbouring *Jeu de Course*, the followers of poor Fred. Archer sped around and around an endless course to make perhaps a dead-heat at the butt. A jibbing mule and a growling bear created any amount of fun, while a gentleman-doll smoked his cigarette with perfect gravity and satisfaction. Messrs. Deverell have very many more amusing mechanical toys, and we can imagine nothing better calculated to keep an audience's attention than an exhibition of this character.

Electrical and Physiological.

Mr. J. B. Thistleton displayed a valuable collection of electrical and physiological instruments. Noteworthy among these were a dead-beat recording galvanometer, which traced movements upon a revolving cylinder with perfect accuracy by the passage of an electrical current through the latter. This tracing may be removed from the cylinder, and kept as a permanent record. The galvanometer may also be used as a reflector, and readings may be taken instantly, as it is perfectly dead beat. Another ingenious instrument was a combined forty-cell sulphate of mercury continuous-current apparatus, with galvanometer, current alternator, combined and reverser, by means of which the Faradaic or galvanic currents may be either used separately or be combined by a single movement of a switch. Mr. Thistleton also showed some continuous-current batteries of various sizes with sulphate of mercury cells; and three sizes of the new portable Faradaic apparatus with chromic acid batteries. Three incandescent electric lamps were lighted by a primary battery of only four small cells. Amongst other novelties were a graduated voltometer, galvanometers, a pocket Faradaic apparatus with sulphate of mercury cells, interrupting handles, and a variety of electrodes, and a galvano-cautery apparatus of the latest pattern. Messrs. R. and J. Beck sent a number of high-power microscopes, beneath which Mr. Thomas Moore, F.R.C.S., illustrated the germ theory by the exhibition of several varieties of the much-talked-of bacilli. The ladies in particular appeared to view with special curiosity the hateful germs which breed zymotic diseases in the human frame. They were, we understand, nearly all "home grown," and the skill with which anthrax, for example, had been developed, under Mr. Moore's fostering care, created some amazement and not a little fright in the beholders. Mr. Moore was present, and seemed well pleased with the interest taken in his bacilli. The Shire Horse Society lent a valuable collection of pictures of horses, and the Autotype Company adorned the walls with some of their really exquisite productions.

